# Supplementary material for: Genome-Wide Expression and Anti-Proliferative Effects of Electric Field Therapy on Pediatric and Adult Brain Tumors
Source: Int J Mol Sci. 2022 Feb 11;23(4):1982. doi: 10.3390/ijms23041982 (PMC8880247; doi:10.3390/ijms23041982)
Supplement: Supplementary file 1 [file ijms-23-01982-s001.zip › Supplement Figure Legends.pdf]

Supplement Figure S1. IC50 curves of TMZ, paclitaxel, and mebendazole for U87-MG, KNS42, SF188, GIN-28, GIN-31, GCE-77, UW228-3, and BXD-1245-EPN cell lines used in the combination experiments. The curves have the higher and lower doses used in the combinational experiments indicated.

Supplement Figure S2. Volcano plots demonstrating the gene expression patterns of up-regulation and down-regulation of GBM cell lines following electrical treatment. KNS42 and GIN-31 cells treated with 200kHz TTFields or 130Hz/10V/450µs electric fields relative to untreated cells.

Supplement Figure S3. Table of top 50 differentially expressed genes from electrically treated cells versus sham controls in GIN-31 and KNS42 cell lines

Supplement Figure S4. KEGG Pathway analysis of the effects of DBS electric fields and TTFields treatment on GBM cell lines. The KEGG Pathways describe the Parkinson Disease, Alzheimer Disease and Huntington Disease pathways. The KEGG Pathways were generated from the gene target lists of commonly differentially expressed genes between the cell lines and treatments. Green boxed genes indicate genes differentially expressed between both DBS and Inovitro treated cell lines and sham treated cells.
