# Supplementary figures and images for: Genome-Wide Expression and Anti-Proliferative Effects of Electric Field Therapy on Pediatric and Adult Brain Tumors
Source: Int J Mol Sci. 2022 Feb 11;23(4):1982. doi: 10.3390/ijms23041982 (PMC8880247; doi:10.3390/ijms23041982)

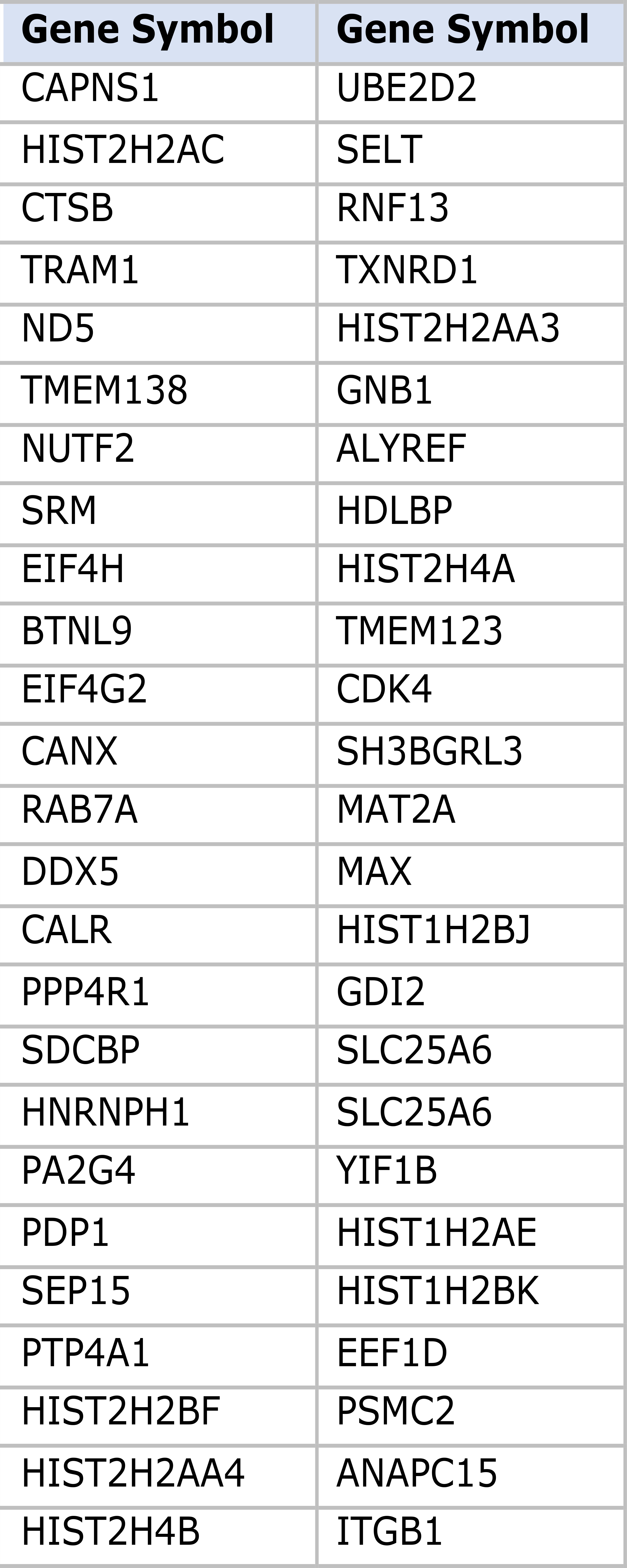

Supplement: Supplementary file 1 [file ijms-23-01982-s001.zip › Supp figure S3.tif]
